# A Clinical Pharmacy Service to Prevent Drug–Drug Interactions and Potentially Inappropriate Medication: A Consecutive Intervention Study in Older Intermediate Care Patients of a Regional Hospital

**DOI:** 10.3390/pharmacy13030060

**Published:** 2025-04-24

**Authors:** Alexander Kilian Ullmann, Oliver Bach, Kathrin Mosch, Thilo Bertsche

**Affiliations:** 1 Clinical Pharmacy Department, Institute of Pharmacy, Medical Faculty, Leipzig University, 04103 Leipzig, Germany; 2 Drug Safety Center, Medical Faculty, Leipzig University, 04103 Leipzig, Germany; 3 Regional Hospital Freiberg gGmbH, 09599 Freiberg, Germany

**Keywords:** pharmacy service, hospital, medication review, drug interactions, potentially inappropriate medication list

## Abstract

*Background:* In intermediate care, older patients with polypharmacy are vulnerable to drug–drug interactions (DDI) and potentially inappropriate medication (PIM). Aims: To perform a consecutive intervention study to evaluate DDI/PIM. *Methods:* Clinically-relevant DDI/PIM were identified using AMeLI (electronic medication list) and PRISCUS 2.0 (PIM list). Consecutive patients (standard care group) were screened for DDI/PIM after admission (t0) and again before discharge (t1). In an interim period, physicians received general education about DDI/PIM. Then, consecutive patients (independent clinical pharmacy group) were screened for DDI/PIM after admission (t2). Physicians were then provided with patient-individualized recommendations by a clinical pharmacist to prevent DDI/PIM. The patients were then screened again for DDI/PIM before discharge (t3). *Results:* In each group, 100 patients were included with data available for evaluation from 97 (standard care group, median age: 78 years [Q25/Q75: 69/84]) and 89 (clinical pharmacy group, 76 years [67/84]). In the standard care group, DDI were identified in 55 (57%) patients after admission (t0) and 54 (56%) before discharge (t1, ARR[t0/t1] = 0.01, NNT[t0/t1] = 100, n.s.). In the clinical pharmacy group, DDI were identified in 32 (36%) after admission (t2; ARR[t0/t2] = 0.21/NNT[t0/t2] = 5, *p* < 0.01) and 26 (29%) before discharge (t3; ARR[t2/t3] = 0.07/NNT[t2/t3] = 15, n.s.; ARR[t1/t3] = 0.27/NNT[t1/t3] = 4, *p* < 0.001). PIM were identified in patients at t0: 34 (35%), t1: 35 (36%, ARR[t0/t1] = −0.01/NNH[t0/t1] = 100, n.s.), t2: 25 (26%, ARR[t0/t2] = 0.09/NNT[t0/t2] = 12, n.s.), t3: 23 (24%, ARR[t2/t3] = 0.11/NNT[t2/t3] = 10, n.s.; ARR[t1/t3] = 0.12/NNT[t1/t3] = 9, n.s.). *Conclusions:* In the standard care group, after admission, many DDI/PIM were identified in older intermediate care patients. Before discharge, their number was hardly influenced at all. General education for physicians led to DDI prevention after admission. In addition, the DDI frequency decreased by providing physicians with patient-individualized recommendations.

## 1. Introduction

According to data from Germany, in 16,000 to 25,000 patients per year, drug-related problems are estimated to cause death with an increased risk due to polypharmacy [1]. Drug-related problems are characterized as events or circumstances in drug therapy that actually or potentially prevent the achievement of the desired therapeutic goals [2]. A quarter of these are considered serious or life-threatening [1]. Drug-related problems not only endanger patient safety but also lead to high costs in the health care system [3]. Patients switching from outpatient to inpatient treatment and vice versa are particularly at risk [4]. This is especially the case for older patients. Due to existing underlying diseases and polypharmacy, older patients are particularly vulnerable to the clinical consequences of drug-related problems. Polypharmacy has been defined differently regarding the number of concurrently taken medications: as the long-term use of two or more medications, as the daily intake of two to three or at least three medications, or as the use of four to five medications. In other studies, the limit for polypharmacy has been at least five medications, over five medications, at least seven, or at least 10 medications [5].

Drug–drug interactions (DDI) occur when one drug adds to or diminishes the effect of another drug (i.e., pharmacodynamic interaction) or affects the absorption, distribution, metabolism, or excretion of another drug (i.e., pharmacokinetic interaction) [6]. DDI cause over a quarter of all adverse drug reactions and are associated with a significant burden on the healthcare system due to increased hospitalizations [6]. The more medications are prescribed, the higher the probability of a clinically relevant DDI [7].

Potentially inappropriate medication (PIM) refers to active ingredients that may be inappropriate for older people and should be avoided. Polypharmacy leads to an increased occurrence of adverse drug reactions, particularly in older patients [8,9,10,11]. There are various expert lists that classified active ingredients as potentially inappropriate, including, for instance, the BEERS [8], PRISCUS [9], EU(7)-PIM [10] and FORTA lists [11]. The PRISCUS list is intended for patients aged 65 and over. It was first published in 2010 and was recently updated in 2022 (PRISCUS list 2.0). The PRISCUS list has the advantage of being tailored to German-speaking countries [12,13].

With the aim of identifying DDI or PIM, medication review and comprehensive interprofessional medication management involving pharmacists has proven to be effective in increasing patient safety [14,15]. This measure can also be carried out in an interprofessional collaboration with physicians (after admission to hospital and before discharge to primary care) leading to potential positive outcomes on hospital re-admissions and other outcomes [16,17,18]. Such an intervention using clinical pharmacy services helps to reduce the occurrence of DDI [19] and PIM [20,21,22].

However, it has been criticized that the quality of studies investigating clinical pharmacy service is often limited and that, as a consequence, more good-quality studies are required. What is more, previous studies are often not transferable to current conditions, in which, for instance, electronic aids such as drug information systems have created new possibilities for precision and time-saving gains in drug information. In addition, much of the published data is restricted to university settings. There has been relatively little research in non-university regional hospitals.

For this reason, the analysis presented here was carried out in such a setting, where clinical pharmacists have not yet been involved in medication reviews or ward supervision. In a consecutive intervention study, the effects on DDI and PIM frequency were evaluated before and after a clinical pharmacy service had been implemented. Additionally, the impact of educating physicians about DDI/PIM prevention was investigated. We hypothesized that DDI and PIM would be common after admission, that standard care would not substantially influence the DDI/PIM frequency before discharge, and that providing education to physicians as well as a patient-individualized clinical pharmacy service would lead to a significant reduction in DDI/PIM frequency.

## 2. Materials and Methods

Patients and setting: From 1 May to 31 October 2024, the study was performed in a regional hospital with specialized care. The hospital provides 335 beds in 12 specialist departments. Over 13,000 patients are treated as inpatients every year.

The study was performed in the hospital’s intermediate care (IMC) unit divided into an internal medicine and a surgical sub-unit. The study was carried out in the internal medicine sub-unit. Here, many older patients were treated, with a focus on patients with cardiovascular diseases.

In the hospital, where the study was performed, pharmacists have not previously been involved in interdisciplinary collaboration with physicians via medication reviews or ward supervision. During this study, an analog patient file was still in use.

Study design: The investigation was performed as a consecutive intervention study in two groups, which retrospectively examined anonymized procedures of routine care.

Study protocol: Two consecutive independent study groups (in which standard care vs. clinical pharmacy service was offered) were evaluated. Patients who had been evaluated once in the first group were subsequently not evaluated a second time, even if they had been re-admitted to the IMC.

- First period (status quo, standard care group): In a first patient group, patient data was evaluated after IMC admission (t0) and before IMC discharge (t1).
- Interim period (between t1 and t2): Physicians were generally educated about DDI/PIM prevention.
- Second period (clinical pharmacy group, patient-individualized clinical pharmacy service offered): In an independent second group of patients, the patient data were analyzed after IMC admission (t2). Patient-individualized information on DDI/PIM prevention was obtained through a medication review performed by a clinical pharmacist (Alexander Kilian Ullmann) and then forwarded to the treating physicians of the IMC unit. Afterwards, the medication list, which was generated for the discharge letter before IMC discharge, was assessed for DDI/PIM (t3).

Inclusion criteria: Patients were included in the evaluation if they were newly admitted to the IMC, were undergoing internal medicine treatment on the IMC unit at the time of the medication review, had at least three active ingredients in their medication list after admission, or were at the outpatient–inpatient interface, i.e., they were admitted externally and were not transferred internally to the IMC unit.

Clinical pharmacy service: To investigate the effect of the clinical pharmacy service, the number of DDI and PIM were evaluated. The clinical pharmacy service was implemented in the consecutive second patient group after the treating physicians (not blinded to treatment or intervention) had been educated about DDI and PIM prevention and the aims of this study. Laboratory values were used for the medication review. However, no direct patient interviews were conducted within this framework. DDI and PIM were identified and classified, then recommendations on how to prevent the identified DDI/PIM in future care were forwarded to the treating physicians in the clinical pharmacy group. All assessments and recommendations were designed by Alexander Kilian Ullmann and reviewed by Oliver Bach.

Outcomes: The number, clinical relevance, and severity of DDI were evaluated and severity was classified into “Contraindicated DDI”, “Serious DDI”, and “Moderate DDI” at t0, t1, t2, and t3 according to the study protocol. The number of identified PIM was assessed at t0, t1, t2, and t3, respectively.

Statistics and data evaluation: To identify DDI, the drug information system AMeLI (Electronic Medication List, Pharmacy of the Clinics of the District of Heidenheim, Germany) was contacted to identify DDI. DDI were classified as clinically relevant according to AMeLI (i.e., those DDI that were at least of moderate severity). Only clinically relevant DDI were further classified into “Contraindicated DDI”, “Serious DDI”, and “Moderate DDI”. The PRISCUS list 2.0 (Witten/Herdecke University) was contacted to identify PIM.

The analysis was performed according to the intention-to-treat principle. This means that all patients included after admission were observed and evaluated until their discharge. However, since this was not a randomized study, but rather an evaluation of routine care processes and measures, it is possible that not all data were available at the end for all initially included patients. These patients could therefore ultimately not be evaluated before discharge. In the clinical pharmacy group, all patients were evaluated, regardless of whether the recommendations were followed or not. The following comparisons were evaluated statistically.

Paired analysis within the study groups:

- t0 vs. t1: McNemar Test to assess the effects of “standard care” on the number of patients with at least one DDI/PIM comparing “after admission” vs. “before discharge” within the standard care group.
- t2 vs. t3: McNemar Test to assess the effects of forwarding “recommendations to prevent DDI/PIM” on the number of patients with at least one DDI/PIM comparing “after admission” vs. “before discharge” within the clinical pharmacy group.

Unpaired analysis between the study groups:

- t0 vs. t2: Chi-Square Test to assess the effects of “educating physicians about DDI/PIM prevention” on the number of patients with at least one DDI/PIM comparing the point in time “after admission” between the standard care group and the clinical pharmacy group.
- t1 vs. t3: Chi-Square Test to assess the effects of forwarding “recommendations to prevent DDI/PIM” on the number of patients with at least one DDI/PIM comparing the point in time “before discharge” between the standard care group and the clinical pharmacy group.

Between the two groups, gender was tested using a Chi-Square Test and age using a Mann–Whitney U Test.

A Point-Biserial Correlation was performed to correlate gender (dichotomous) with the frequency of DDI and PIM, and a Spearman’s Rho Correlation to correlate patients’ age with the frequency of DDI and PIM.

A Chi-Square Test was performed to test for the eight most frequent diagnosis groups (number of patients with at least one diagnosis in the respective group). After this test a Bonferroni correction was performed for multiple testing (*n* = 8) with a *p* ≤ 0.006 indicating significance.

Data is presented as absolute number and frequencies in percent. Absolute risk reductions (ARR) as well as the number needed to treat (NNT) and the number needed to harm (NNH) were additionally calculated. The statistical tests were performed with Social Science Statistics free resources for students and researchers (Available online: https://www.socscistatistics.com/, last accessed on 15 April 2025) and MedCalc Statistical software package for biomedical research (Available online: https://www.medcalc.org/calc/mcnemar.php, last accessed on 15 April 2025).

Sample size calculation: Based on recent literature [23] and pretests in the setting, the sample size was calculated. We predicted that in 45% (*p*1 = 0.45) of the patients, at least one clinically relevant moderate DDI would occur in the first group. A reduction to at least 25% (*p*2 = 0.25) due to the clinical pharmacy service in an independent group was considered clinically relevant. Assuming the results of this range, a two-sided test at a significance level of *α* = 0.05 and a power of 1 − *β* = 0.80 would provide a sample size of about 98 per group for this outcome. All patients included in the study were analyzed according to their allocation (intention-to-treat analysis). All analyses were performed in the originally assigned groups. A *p* ≤ 0.05 was considered significant (Bonferroni correction for multiple testing in diagnoses groups (*n* = 8) with a significance of *p* ≤ 0.006). The power calculation was performed by the web calculator of the University of Cologne, Faculty of Medicine, Institute of Medical Statistics and Bioinformatics: Calculation of power and sample size for proportions (relative frequencies) of two independent samples (Available online: https://imsiewebarchiv.uni-koeln.de/beratung/rechner/ps.html, last accessed on 15 April 2025).

## 3. Results

### 3.1. Patient Characteristics

We initially included 100 patients per group according to the sample size calculation. Due to prematurely discharged patients and missing data, from the first group (standard care group), 97 patients were finally evaluated. From the second group (clinical pharmacy group), 89 patients were finally evaluated. Of the 97 in the standard care group, 51 (53%) were male vs. 51 out of 89 (57%) in the clinical pharmacy group (n.s.). The median age was 78 (Q25; Q75: 69; 84) vs. 76 (67; 84) years (n.s.). Patient characteristics for both groups are summarized in Table 1.

### 3.2. Drug–Drug Interactions (DDI)

As presented in Table 2, in the standard care group, 481 DDI were identified in 97 patients (4.96 DDI per patient) after admission (t0) according to AmeLI. Of those 481 DDI, 118 (25%) were classified as clinically relevant (1.21 clinically relevant DDI per patient). Fifty-five (57%) patients were affected by at least one clinically relevant DDI. Of 118 clinically relevant DDI, 8 (7%) DDI were classified as contraindicated, 34 (29%) as serious and 76 (64%) as moderate DDI.

In the standard care group, 465 DDI were identified in 97 patients (4.79 DDI per patient) before discharge (t1). Of those 465 DDI, 94 (20%) were classified as clinically relevant (0.97 clinically relevant DDI per patient). Fifty-four (56%) patients were affected by at least one clinically relevant DDI. Of those 94 clinically relevant DDI, five (5%) were classified as contraindicated, 32 (34%) as serious, and 57 (61%) as moderate.

In the clinical pharmacy group, a total of 327 DDI were identified in 89 patients (3.67 DDI per patient) after admission (t2) according to AmeLI. Of those 327 DDI, 69 (21%) were classified as clinically relevant (0.76 clinically relevant DDI per patient). Thirty-two (36%) patients were affected by at least one clinically relevant DDI. Of 69 clinically relevant DDI, two (3%) were classified as contraindicated, 26 (38%) as serious and 41 (59%) as moderate DDI.

In the clinical pharmacy group, 307 DDI were identified in 89 patients (3.45 DDI per patient) before discharge (t3). Of those 307 DDI, 36 (12%) were classified as clinically relevant (0.40 clinically relevant DDI per patient). Twenty-six (29%) patients had at least one clinically relevant DDI. Of those 36 clinically relevant DDI, none (0%) were classified as contraindicated, seven (19%) as serious and 29 (81%) as moderate DDI.

ARR and NNT were calculated as follows: ARR[t0/t1] = 0.01, NNT[t0/t1] = 100 (n.s.), ARR[t0/t2] = 0.21, NNT[t0/t2] = 5 (*p* < 0.01), ARR[t2/t3] = 0.07, NNT[t2/t3] = 15 (n.s.), and ARR[t1/t3] = 0.27, NNT[t1/t3] = 4 (*p* < 0.001).

The clinical severity of the DDI (assessed according to AMeLI) is presented in Table 1. Contraindicated DDI in the standard care group included the combinations of xaban and certoparin (increased risk of bleeding), xaban and heparin (increased risk of bleeding), atorvastatin and erythromycin (increased risk of myopathy and/or rhabdomyolysis), amiodarone and metoprolol (increased risk of hypotension, bradycardia or cardiac arrest), ivabradine and verapamil (increased risk of severe bradycardia), and solifenacin and erythromycin (increased risk of severe anticholinergic effects). Contraindicated DDI in the clinical pharmacy group included simvastatin and erythromycin (increased risk of rhabdomyolysis). In the serious DDI, the risks of myopathy, bleeding, and sedation were increased. In the moderate DDI, the most frequent risks were hypomagnesemia, reduced efficacy, renal impairment, and hyperkalemia.

A correlation of the frequency of DDI with age was found only at t0 (r = 0.27683, *p* < 0.01). All other points in time showed no statistically significant correlation. There was no correlation of the frequency of DDI with gender.

In the standard care group, no DDI were life-threatening and had to be reported to the responsible physicians as an “emergency intervention”.

### 3.3. Potentially Inappropriate Medications (PIM)

As presented in Table 3, in the standard care group, 44 PIM were identified in 34 patients (0.45 PIM per patient, 35% of the patients were affected by at least one PIM) after admission (t0) according to PRISCUS 2.0. Before discharge (t1), 49 PIM were identified in 35 patients (0.51 PIM per patient, 36% affected patients).

In the clinical pharmacy group, 31 PIM were identified in 25 patients (0.35 PIM per patient, 28% of the patients were affected by at least one PIM) after admission (t2) according to PRISCUS 2.0. Before discharge (t3), 27 PIM were identified in 23 patients (0.30 PIM per patient, 26% of the patients were affected by at least one PIM).

ARR and NNT were calculated as follows: ARR[t0/t1] = −0.01, NNH[t0/t1] = 100 (n.s.), ARR[t0/t2] = 0.09, NNT[t0/t2] = 12 (n.s.), ARR[t2/t3] = 0.11, NNT[t2/t3] = 10 (n.s.), and ARR[t1/t3] = 0.12, NNT[t1/t3] = 9 (n.s.).

The specific drug groups most frequently included in PIM were the following: proton pump inhibitors (PPI), sedatives, anticholinergics, antihypertensives, and opioids.

The number of PIM was correlated with age at all time points (t0: r = 0.29596, *p* < 0.01; t1: r = 0.24294, *p* < 0.05; t2: r = 0.27247, *p* < 0.01; t3: r = 0.3136, *p* < 0.01). A significant correlation with gender (for the characteristic “women”) was found for PIM only in the clinical pharmacy group at t2 (r = 0.21767, *p* < 0.05) and at t3 (r = 0.22647, *p* < 0.05).

In the first patient group, no PIM were life-threatening and had to be reported to the responsible physicians as an “emergency intervention”.

### 3.4. Recommendations in the Clinical Pharmacy Group

The recommendations forwarded to treating physicians in the clinical pharmacy group related to discontinuation of medication, change of medication, starting a new medication, change in strength of a medication, change in dosage of a medication, and other measures. Several possibilities were possible at the same time. Eighty-two percent of all individualized clinical pharmacy service recommendations were accepted by the physicians in the clinical pharmacy group.

## 4. Discussion

### 4.1. Key Results

We evaluated older intermediate care patients in a regional hospital in two independent patient groups with approximately half of the patients in each group having cardiovascular (primary) diagnoses. Neurological, pulmonary, gastroenterological, infectious, metabolic (except for diabetes), diabetological, and nephrological (co-)diagnoses were numerous (no significant differences between the study groups except for a neurology diagnosis).

Our study indicates that DDI and PIM were alarmingly frequent. As expected, PIM occurred more frequently with increasing age in all groups and at all time points. PIM correlated with female gender in the clinical pharmacy group at t2 and t3. A total of 461 DDI were found in the standard care group. Even if only those DDI that are clinically relevant were considered, a considerable number of 118 were identified. This means that 1.21 clinically relevant DDI occurred per patient and 57% of patients were affected by at least one clinically relevant DDI. When offering standard care, the number of clinically relevant DDI (i.e., those that were of at least moderate severity) and PIM were not affected in a clinically relevant way.

After physicians had been educated about DDI and PIM in general before the clinical pharmacy service was introduced into routine care, awareness of the topic of DDI (and PIM) substantially increased. This can be suspected since physicians were openly more concerned about DDI (and PIM) in the context of their admission management (without pharmaceutical involvement). Comparing the number of patients with at least one DDI or PIM in the second group with the number in the first, a NNT of 5 (significant) was achieved for DDI and 12 for PIM (not significant), comparing the standard care group with the clinical pharmacy group. This cannot be explained by differences in patient characteristics, as age, gender, and diagnoses can be described as very similar. The patient-individualized clinical pharmacy service intervention was carried out in a second period, and the better initial level after admission between the standard care and the clinical pharmacy group indicates that general education for physicians about DDI/PIM prevention had a high influence. This instruction obviously increased the awareness of the participating physicians about DDI (and PIM). Apparently, this general step before the implementation of the clinical pharmacy service has already led to an attentive switch of medication to the hospital formulary, with some DDI (and PIM) already being eliminated by the physicians themselves. However, in the context of routine implementation, this non-specific effect, which actually precedes the clinical pharmacy service in terms of increased awareness among physicians, may well be desirable.

When the patient-individualized recommendations by a pharmacist were then carried out by the physicians, a further improvement was achieved in terms of the number of patients affected by at least one DDI: a comparison between the standard care group and the clinical pharmacy group at the time before discharge from the hospital showed a NNT of 4 for DDI (significant) and 9 for PIM (not significant).

The results were particularly impressive with regard to the sub-categories, i.e., contraindicated, serious or moderate DDI. For example, the contraindicated DDI were completely prevented at the end. The overall positive effects at the end of the study (t3) can likely be regarded as a combined effect of the general and patient-specific strategies.

### 4.2. Interpretation

DDI occurred in over 50% of hospitalized older patients with cardiovascular disease and polypharmacy [24]. With 57% of patients affected before the introduction of any pharmaceutical services, our frequencies were comparable to these published data. According to the literature, however, this number continued to rise upon discharge from the hospital. This underlines the need for strategies to be considered in hospital, including for post-hospital care, to reduce the number of hospital readmissions [25]. In contrast to those literature reports, we did not carry out a follow-up in primary care after discharge as part of our present study. The following were named in the literature as strategies of a clinical pharmacy service [25]: patient needs assessment, medication reconciliation, patient education, arranging timely outpatient appointments, and providing telephone follow-up. Our service differed from those strategies as we offered medication review with feedback recommendation to the treating physicians as a clinical pharmacy service tool. Corresponding databases are often used to identify DDI [26]. However, there is skepticism in the literature about their benefit in practical use, at least when they are used on their own without appropriate implementation or professional support [26]. The fact that the information frequently differs also plays a key role and first requires a professional assessment before specific, clear recommendations for clinical management can be derived [27]. However, one reason for difficulties in the evaluation of DDI in databases is the lack and quality of data from clinical studies [28]. We only contacted one database (i.e., AMeLI) as part of this clinical pharmacy service. We consider the research in this database to be extremely profound, but we concede that a detailed analysis of primary data could certainly have further improved the quality. However, this is certainly hardly practical under the personal and organizational conditions of a smaller institution, so we have decided on this procedure. As shown in [23], the relative risk of a patient experiencing at least one DDI-related adverse event decreased from 60 (44%) patients in the control group to 32 (25%) in the intervention group. Those events included some serious events such as QT(C) prolongation, which were substantially reduced. We also found a considerable and significant reduction due to our standard care and additional effects from our clinical pharmacy service. However, it should be borne in mind that we used a consecutive design and did not utilize a randomized control, which is better to reduce bias and confounding factors.

Current data indicate a reduction in PIM prevalence since 14.5% of German patients aged ≥ 65 years were prescribed at least one PIM per year in 2019, while the number was 24% in 2009 [9]. However, there is criticism of the PIMs, e.g., with regard to the lack of information on appropriate alternatives [29]. Therefore, the clinical management of PIM requires not only drug information, but also integration into the clinical pharmacy service to deliver appropriate recommendations for clinical use [30]. Of interest in comparison with our results is a study in [31], which found no demonstrably significant change in prevalence between admission and discharge from an IMC setting. However, unlike us, the authors included not only potentially inappropriate medications (PIM) but also potential missed prescriptions (PPO), referred to as potentially inappropriate prescriptions (PIP). In [32], the knowledge of physicians and pharmacists about PIM criteria was investigated. The authors conclude that an interprofessional approach, such as the one we used in our study, is recommended. What is more, the effects from a pharmacoeconomic perspective might also be very relevant, as reported in [33].

### 4.3. Generalizibility

At the time of this evaluation, only patient files in paper format were available for the study, which might limit generalizability. Modern electronic availability could have considerably optimized the practicability, the time required and the completeness of the data collection according to previous experience with such framework conditions.

In terms of confirmatory studies improving external validity, larger numbers of cases are required for scientific investigation, especially for less frequently occurring drug-related problems such as PIM.

Nevertheless, we believe that we can conclude from our evaluation, which focuses on defined clinically-relevant and well-established factors, that the clinical pharmacy service concept has also proven to be effective in principle in a non-university environment and should therefore be transferable to other areas and clinics.

### 4.4. Future Considerations

The data suggest that the clinical pharmacy service concept should be continued and expanded to other wards. It may be useful to expand or adapt the concept to other clinically relevant drug-related problems that could be of interest to the respective patient group. Future studies should then also include follow-up on peripheral units or before discharge to the home setting or nursing home.

The positive effects of combining general and patient-individualized strategies in the clinical management of DDI/PIM should also provide important guidance for future implementation of the clinical pharmacy service. As a consequence, patient-individualized recommendations to physicians should always be preceded by the training of those physicians, as training already plays a significant role in the success of the clinical pharmacy service.

### 4.5. Benefit-Risk Assessment

As this is an evaluation of processes that were routinely implemented in the hospital, there is no additional risk from the evaluation. Furthermore, the introduction of the clinical pharmacy service is not expected to pose any risk to patients, as even in the event of inadequate recommendations, the decision on patient treatment remains with the treating physician. On the contrary, the results show that any clinical pharmacy service has the potential to reduce the risk for DDI (and PIM).

### 4.6. Limitations and Sources of Potential Bias and Confounders

The following items should be considered when evaluating the results of this study.

First, the investigation of the clinical pharmacy service was performed in a consecutive study design without randomization.

Second, the study was performed in only one hospital. To what extent the results of this setting would be transferable to other settings with other clinical focuses outside of IMC, and to older patients with a cardiology focus, should be critically considered.

Third, the pharmaceutical recommendations were dependent on the physicians for implementation. The acceptance rate of 82% appears good. However, this means that a not inconsiderable proportion of almost one fifth of the clinical pharmacists’ recommendations remained unconsidered by the treating physicians.

Fourth, we did not investigate the sustainable implementation of the recommendations by the general practitioner and specialist in long-term observation after discharge.

Fifth, although the potential clinical risks of identified DDI were considered, actual clinical events were not investigated.

## 5. Conclusions

A high prevalence of drug–drug interactions (DDI) and potentially inappropriate medication (PIM) was identified in older patients with polypharmacy after IMC admission. Standard care did not lead to any relevant changes in DDI/PIM frequency between IMC admission and discharge. Providing physicians with general education about DDI/PIM prevention had a measurable effect on the physicians’ awareness of DDI during patients’ admission management. In addition, patient-individualized recommendations by a clinical pharmacist to treating physicians succeeded in preventing DDI.

The results suggest that the topic of DDI/PIM management should be addressed to physicians in general for each individual patient in IMC settings, with special regard to regional hospitals.

## 6. Impact of Findings on Practice Statements

What is known:

- Drug–drug interactions (DDI) and potentially inappropriate medication (PIM) jeopardize patient safety.
- Patients of advanced age and in intermediate care facilities are among the most vulnerable patient groups with clinical consequences of DDI and PIM.
- Status quo studies and solution strategies concerning DDI and PIM are mostly published in university settings.

What is new:

- DDI and PIM were found to be common in older intermediate care patients in a regional hospital.
- Standard care did not lead to any relevant influence on DDI/PIM frequency.
- General education for physicians about DDI/PIM led to physicians paying more attention to DDI in admission management and, as a consequence, to less frequent DDI after admission.
- Providing physicians patient-individualized recommendations within a clinical pharmacy service decreased DDI frequency before discharge.

## Figures and Tables

**Table 1:** Patient characteristics. Significance for gender and age was not adjusted with a significance of *p* ≤ 0.05. Significance for diagnosis is presented after Bonferroni correction (*n* = 8) for multiple testing with *p* ≤ 0.006. n.s.: not significant; n.a.: not assessable (sample size too small). Patients with at least one diagnosis in the corresponding main diagnosis group were analyzed. Multiple answers were possible only between different diagnosis groups.

	Standard Care Group	Clinical Pharmacy Group	Statistics
**Patients**			
included for evaluation	100	100	
with full data available	97	89	
**Gender**			n.s.
Female	46 (47%)	38 (43%)	
Male	51 (53%)	51 (57%)	
**Age**			n.s.
Median (Q25; Q75) [years]	78 (69; 84)	76 (67; 84)	
**Primary diagnoses in the** **following disciplines** **(percent of patients affected)**			
Cardiology	54 (56%)	44 (49%)	n.s.
Neurology	44 (45%)	12 (13%)	*p* < 0.001
Pulmonology	15 (15%)	19 (21%)	n.s.
Gastroenterology	9 (9%)	18 (20%)	n.s.
Infectiology	8 (8%)	7 (8%)	n.s.
Metabolics (other than Diabetes)	7 (7%)	8 (9%)	n.s.
Diabetology	6 (6%)	9 (10%)	n.s.
Nephrology	5 (5%)	13 (15%)	n.s.
Urology	3 (3%)	0 (0%)	n.a.
Toxicology	2 (2%)	8 (9%)	n.a.
Oncology	2 (2%)	3 (3%)	n.a.
Orthopaedics/Trauma Surgery/Rheumatology	2 (2%)	3 (3%)	n.a.
Psychiatry	1 (1%)	2 (2%)	n.a.
Dermatology/Allergology	0 (0%)	2 (2%)	n.a.

**Table 2:** Drug–drug interaction (DDI) assessment. First period (status quo, standard care group): Patient data assessment in the first patient group (*n* = 97) after IMC admission (t0) and before IMC discharge (t1). Interim period without data assessment (between t1 and t2): physicians were given general education about DDI/PIM prevention. Second period (clinical pharmacy group, patient-individualized clinical pharmacy service offered): Offering patient-individualized information about DDI/PIM prevention to the treating physicians after medication review by a clinical pharmacist. Patient data assessment in a second (independent from the first) patient group (*n* = 89) after IMC admission (t2) and before IMC discharge (t3). ^a^ classified according to AmeLI; ^b^ by at least one clinically relevant DDI; IMC: intermediate care (unit). ARR: Absolut risk reduction; NNT: number needed to treat. Significant with *p* ≤ 0.05; n.s.: not significant.

Patient Group	Project Period	All DDI	Clinically Relevant DDI ^a^	Contraindicated DDI ^a^	Serious DDI ^a^	Moderate DDI ^a^	Statistics
**First period** Standard care group (*n* = 97)	**t0:** First medication list after IMC admission	***n* = 481** 4.96 per patient	**118 (25%)** 1.21 per patient, 55 (57%) patients affected ^b^	**8** (7%)	**34** (29%)	**76** (64%)	-
	**t1:** Last medication list before IMC discharge	***n* = 465** 4.79 per patient	**94 (20%)** 0.97 per patient, 54 (56%) patients affected ^b^	**5** (5%)	**32** (34%)	**57** (61%)	**ARR (t0 vs. t1)** 0.57 − 0.56 = 0.01 [NNT: 100] n.s.
**Second period** Clinical pharmacy group (*n* = 89)	**t2:** First medication list after IMC admission	***n* = 327** 3.67 per patient	**69 (21%)** 0.78 per patient, 32 (36%) patients affected ^b^	**2** (3%)	**26** (38%)	**41** (59%)	**ARR (t0 vs. t2)** 0.57 − 0.36 = 0.21 [NNT: 5] *p* < 0.01
	**t3:** Last medication list before IMC discharge	***n* = 307** 3.45 per patient	**36 (12%)** 0.40 per patient, 26 (29%) patients affected ^b^	**0** (0%)	**7** (19%)	**29** (81%)	**ARR (t2 vs. t3)** 0.36 − 0.29 = 0.07 [NNT: 15] n.s. **ARR (t1 vs. t3)** 0.56 − 0.29 = 0.27 [NNT: 4] *p* < 0.001

**Table 3:** Potentially inappropriate medication (PIM) assessment. First period (status quo, standard care group): Patient data assessment in the first patient group (*n* = 97) after IMC admission (t0) and before IMC discharge (t1). Interim period without data assessment (between t1 and t2): physicians were given general education about DDI/PIM prevention. Second period (clinical pharmacy group, patient-individualized clinical pharmacy service offered): Offering patient-individualized information about DDI/PIM prevention to the treating physicians after medication review by a clinical pharmacist. Patient data assessment in a second (independent from the first) patient group (*n* = 89) after IMC admission (t2) and before IMC discharge (t3). ^a^ classified according to PRISCUS 2.0; ^b^ by at least one PIM; IMC: intermediate care (unit). ARR: Absolute risk redutcion; NNT: number needed to treat; NNH: number needed to harm. Significant with *p* ≤ 0.05; n.s.: not significant; n.a.: not assessable (sample size too small).

Patient Group	Project Period	PIM ^a^	Statistics
**First period** Standard care group (*n* = 97) With	**t0:** First medication list after IMC admission	**44** 0.45 per patient, 34 (35%) patients affected ^b^	-
	**t1:** medication list before IMC discharge	**49** 0.51 per patient, 35 (36%) patients affected ^b^	**ARR (t0 vs. t1)** 0.35 − 0.36 = −0.01 [NNH: 100] n.s.
**Second period** Clinical pharmacy group (*n* = 89)	**t2:** First medication list after IMC admission	**31** 0.35 per patient, 25 (28%) patients affected ^b^	**ARR (t0 vs. t2)** 0.35 − 0.26 = 0.09 [NNT: 12] n.s.
	**t3:** medication list before IMC discharge	**27** 0.30 per patient, 23 (26%) patients affected ^b^	**ARR (t2 vs. t3)** 0.35 − 0.24 = 0.11 [NNT: 10] n.a. **ARR (t1 vs. t3)** 0.36 − 0.24 = 0.12 [NNT: 9] n.s.

## Data Availability

The data presented in this study are available on request from the corresponding author, insofar as this is justifiable for ethical and data protection reasons.
